# Hotgenes: an R package for reducing bottlenecks in bulk omics data exploration and collaboration

**DOI:** 10.1093/bioinformatics/btag434

**Published:** 2026-06-24

**Authors:** Richard Virgen-Slane

**Affiliations:** Drug Safety R&D, Pfizer Inc, San Diego, CA, United States

## Abstract

**Summary:**

A critical part of omics analysis is the transition from early data exploration to final interpretation, often including different analytical platforms and the proliferation of figures, tables, and files. To minimize potential errors and delays that can occur during this process, we have developed an R package called “Hotgenes” that contains a wide range of flexible utilities available in a single modular Shiny application. With Hotgenes, differential expression results generated from bulk omics platforms can be imported and shared among collaborators with minimal coding. Furthermore, the modular Hotgenes user interface can be customized by advanced users to fit the needs of their evolving pipelines.

**Availability and Implementation:**

Hotgenes is implemented in R and is freely available at https://github.com/pfizer-opensource/Open-Hotgenes. A permanent archived version is available at https://doi.org/10.5281/zenodo.20129460.

## 1 Introduction

With increasing access to streamlined omics platforms, there is a higher demand for investigators to organize, analyze, and translate complex data into meaningful biological mechanism(s). This process becomes further complicated by collaborative studies that require integration of different endpoints from a collection of flat files. Specialized data analysis systems, such as Illumina’s BaseSpace for transcriptomics and Galaxy (Galaxy 2024) for a wide range of omics applications, provide investigators with analytical tools accessible via a web browser. These methods provide a means for sharing projects with collaborators; however, they do require account setup and extensive training for collaborators new to omics analysis.

The release of R Shiny has enabled the development of open-source R tools, such as NASQAR (Yousif *et al.* 2020), which provides native support for intuitive web-based user interfaces to transcriptomics analysis packages like DESeq2 (Love *et al.* 2014) or Seurat (Satija *et al.* 2015). Another R package, GLIMMA (Kariyawasam *et al.* 2021), supports direct import from popular differential expression analysis methods like limma (Ritchie *et al.* 2015), and edgeR (Robinson *et al.* 2010), in addition to DESeq2.

While the tools above allow investigators to carry out advanced differential expression analysis and deliver results as dynamic reports to collaborators, they are limited to specific differential expression algorithms and do not offer user interfaces supporting integration of auxiliary assay data. Additionally, limitations in data exploration features often compel collaborators to use software like Excel, which can introduce errors (Zeeberg *et al.* 2004). To fill this gap, we developed *Hotgenes*, an R package that supports downstream exploration and interpretation of differential expression results generated from bulk omics data, with integrated support for auxiliary assay data. Auxiliary assay data refer to additional sample‑level measurements that are not part of the differential expression model itself, such as phenotypic annotations, clinical chemistry values, pathology scores, or other orthogonal assay readouts, which can be integrated for downstream interpretation and visualization.

Our goal was to create Hotgenes as a platform streamlined for all levels of omics experience, while maintaining the ability to support custom pipeline development that sometimes drives collaborations. To this end, we defined a new S4 object, called Hotgenes, that is constructed from completed differential expression or regression‑based analyses and simplifies downstream exploration across diverse bulk omics data types. This object supports integration of auxiliary assay data, feature alias mapping, and streamlines Gene Set Variation Analysis (GSVA) (Hanzelmann *et al.* 2013). Furthermore, the original data object imported into Hotgenes is readily accessible should the analysis need refinement. Hotgenes provides native support for importing results from DESeq2, limma, and DRomics (Larras *et al.* 2018), enabling downstream exploration of both standard differential expression and dose–response analyses while preserving the original upstream models. Detailed descriptions of the Hotgenes data model and API are provided in the Supplementary Methods, available as supplementary data at *Bioinformatics* online.

When deployed using the Hotgenes Shiny application, collaborators can use the provided modules to browse results by volcano plot, Venn diagram (with directionality), or by clustering on principal components with available auxiliary assay data using the FactoMineR package (Lê *et al.* 2008). The embedded Gene Set Enrichment Analysis (GSEA) module is powered by the fgsea package (Korotkevich *et al.* 2021) and uses gene sets provided by the msigdbr package (Liberzon *et al.* 2011), and can also accept custom gene sets as well (Elcombe *et al.* 2025). The persistent Hotgenes data object facilitates straightforward downstream visualization of bulk RNA‑seq results in independent studies, including principal component analysis and heatmap‑based summarization with associated sample metadata (Ji *et al.* 2025, Hwang *et al.* 2026).

## 2 Shiny Hotgenes

We wrote the Hotgenes user interface as a collection of Shiny modules. This means that the individual tabs in the user interface can function independently to support custom application development by advanced users. However, as described below, we designed these modules to work in concert for enhanced user experience and utility.

**Figure 1:**
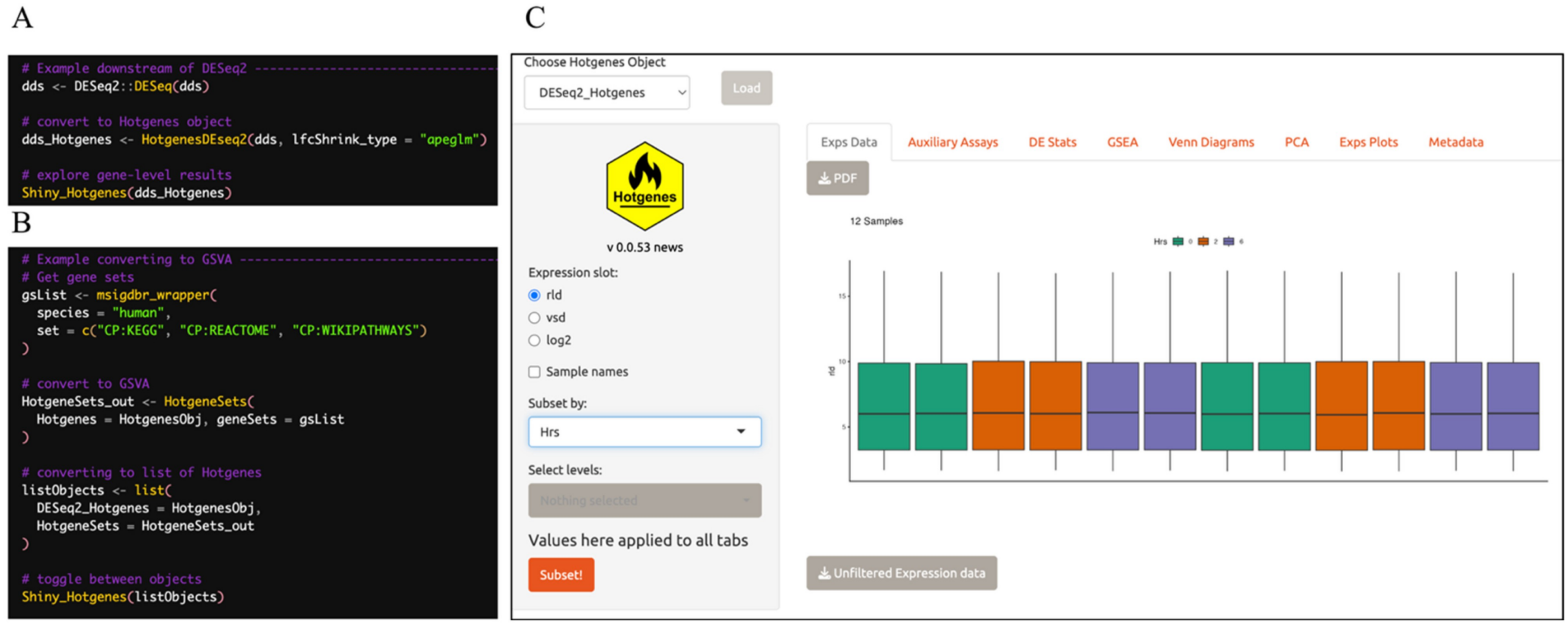
Overview of the Hotgenes workflow and Shiny user interface. (A) Example code showing creation of a Hotgenes object downstream of a DESeq2 analysis. (B) Conversion of gene-level results to pathway-level scores using GSVA. (C) Example session of the Shiny Hotgenes user interface illustrating interactive downstream exploration of differential expression results.

The complete Hotgenes user interface can be accessed using the Shiny_Hotgenes() function, which accepts either a single Hotgenes object or a named list of Hotgenes objects (Fig. 1B). When a named list of Hotgenes objects is passed to Shiny_Hotgenes(), users will be able to switch between the different objects (Fig. 1C). This is useful when reviewing results generated using distinct methods, such as gene-level analysis versus GSVA. Additionally, if Shiny_Hotgenes() is deployed as an application hosted by Posit connect, multiple sessions of the same application can be reviewed in parallel.

## 3 Shiny Hotgenes: check and update data

The “Auxiliary assays” tab allows users to review available assays and generate templates to simplify updates to the auxiliary_assays slot. In this tab, collaborators may upload formatted auxiliary assays into the application to verify alignment with available data, and preview results generated in the “PCA” and “Exps Plot” tabs. However, any new data uploaded into the Shiny application should be considered transient as they are not retained after the session closes. This can be solved by updating the Hotgenes object in R with the auxiliary_assays() function and then redeploying the application.

The “Exps data” tab is a direct interface with the Normalize_Expression slot. Users can select available omics data, subset samples by variables provided in the coldata slot, and review dynamic boxplots of the selected data. This tab controls the omics data used by the other tabs.

## 4 Shiny Hotgenes: identify features of interest

The “DE Stats” tab allows users to summarize all contrast results with dynamic tables, summary plots, interactive volcano plots, and heatmap. This tab allows users to set the threshold for significance and review statistics across contrast for features of interest. Aliases provided in the Mapper slot are dynamically merged with content generated in this tab.

With the GSEA tab, users can pick a contrast of interest, select gene sets, specify the appropriate column from the Mapper slot for alias mapping, and species. This tab provides hyperlinks to GSEA and fgsea package resources to facilitate interpretation. Enrichment results can be exported, along with heatmaps with top features driving signatures.

In the PCA tab, expression data set by the “Exps data” tab can be clustered using principal components. However, only a subset of this data is used, which is controlled by the contrasts and thresholds set by the user. Users may also select features from the coldata and auxiliary_assays slots to be merged with the expression data as supplementary data, available as supplementary data at *Bioinformatics* online. This tab returns plots and tables summarizing the features linked to the detected clusters. Clustered features can be reviewed in a customizable heatmap and will be available for enrichment analysis in the GSEA tab.

Finally, the “Venn Diagram” tab provides a simple method for visualizing logical relationships between features and up to four contrasts. When directionality is enabled, features from each contrast are subdivided by contrast name appended with “up” or “down” based on the direction of change. Like the other tabs, features identified by the tab can be visualized by a heatmap.

## 5 Shiny Hotgenes: review and share

Both the coldata and Mapper slots can be reviewed as dynamic tables in the “Metadata” tab. This allows collaborators to review study design variables and inspect feature alias mapping used for GSEA.

When features of interest are identified, relationships can be reviewed in the “Exps Plot” tab, which generates plots from the coldata, auxiliary_assays, and Normalize_Expression slots. Plots are interactive, allowing inspection of individual data points. Additionally, many options for generating custom figures are provided, such as factor level reordering and facet selection. Although this tab provides convenient plots, formatted data is also returned so users may generate refined figures using software of their choice.

Detailed, annotated walkthroughs of the Shiny interface, including representative analysis workflows demonstrating iterative prioritization of candidate genes across differential expression, multivariate structure, contrast overlap, and pathway-level interpretation, are provided as vignettes in the public GitHub repository.

## 6 Limitations and user support

Hotgenes operates exclusively at the downstream stage of bulk omics data analysis and does not provide native functionality for performing differential expression modeling. Single‑cell RNA‑sequencing workflows and integrated multi‑omics modeling are not currently supported beyond representation of additional assays as auxiliary sample‑level data. While the Shiny interface allows exploration of multiple Hotgenes objects provided as a named list, equivalent functionality is not yet exposed through the command‑line API.

The source code, documentation, and annotated vignettes are maintained in the public GitHub repository. Users are encouraged to fork the repository and submit pull requests for contributions, and usage questions may be directed to the corresponding author.

## 7 Future directions

Ongoing development of Hotgenes focuses on improving interoperability and usability while preserving the separation between upstream statistical modeling and downstream data exploration. Planned directions include extending programmatic interfaces for working with multiple Hotgenes objects, enhancing support for standardized result formats produced by additional differential expression frameworks, and improving integration with upstream workflows through well‑defined APIs. These efforts aim to further streamline collaborative exploration of bulk omics results without introducing new modeling assumptions or duplicating functionality provided by established analysis tools.

## 8 Conclusions

We developed a modular Shiny application to organize, streamline, and simplify omics data analysis for collaborators. To achieve this, we have built an R object to unify data from different omics platforms, ensuring compatibility with downstream methods, and facilitating integration of auxiliary assay data. Our framework offers flexibility, which can be modified by advanced users in the R community to develop custom applications.

## Supplementary Material

btag434_Supplementary_Data

## Code availability statement

The code described in this manuscript is available in the GitHub repository: https://github.com/pfizer-opensource/Open-Hotgenes. This repository contains all scripts, example data, and documentation necessary to explore the functionality described in the manuscript. Detailed installation instructions, system requirements, and package dependencies are provided in the repository README. A permanent archived version of the code corresponding to this manuscript is available at Zenodo: https://doi.org/10.5281/zenodo.20129460. For any questions or issues related to the code, please contact the corresponding author. Hotgenes is distributed under the Apache 2.0 license.
