# Supplementary material for: Hotgenes: an R package for reducing bottlenecks in bulk omics data exploration and collaboration
Source: Bioinformatics. 2026 Jun 24;42(7):btag434. doi: 10.1093/bioinformatics/btag434 (PMC13371767; doi:10.1093/bioinformatics/btag434)
Supplement: btag434_Supplementary_Data [file btag434_supplementary_data.docx]

**Supplementary Methods**

This Supplementary Methods document provides detailed descriptions of the Hotgenes internal data model and application programming interface (API), which are summarized at a high level in the main manuscript.

**Support for DRomics-based dose-response analyses**

Hotgenes supports dose–response analyses performed using the DRomics framework by providing a dedicated constructor function ($HotgenesDRomics()$). Results generated by DRomics, including benchmark dose calculations and associated statistics, are imported into a Hotgenes object in the same standardized format used for DESeq2- and limma-based analyses. This allows DRomics results to be explored alongside normalized expression data, sample metadata, auxiliary assay data, and downstream analyses such as PCA, GSVA/ssGSEA, and pathway-level interpretation. As with other supported frameworks, Hotgenes does not re‑fit dose–response models but preserves and exposes the original DRomics outputs for consistent downstream exploration.

**Hotgenes object: introduction**

Users can create a Hotgenes object by simply importing flat files into R and passing contents (requirements described below) to the $HotgenesUniversal()$ function. Alternatively, we wrote $HotgenesDEseq2(), Hotgeneslimma()$, and $HotgenesDRomics()$ functions to create Hotgenes objects directly from DESeq2 (Figure 1A), limma, and DRomics objects, respectively. To ensure compatibility across differential expression algorithms with downstream analysis, we designed the Hotgenes object with key slots for standardizing methods (described in the sections below), enabling streamlined conversion of gene-level Omics data into GSVA via the $HotgeneSets()$ function (Figure 1B).

**Hotgenes object: differential expression methods slots**

The $Output\_DE$ slot contains results from each contrast as data frames within a named list. Each data frame contains the following: feature identity, average expression, log2 foldchange, test statistic, p-value, and adjusted p-value (labeled as: “Feature”, “baseMean”, “log2FoldChange”, “stat”, “pvalue”, and “padj”). Although additional columns may be provided during object creation, we do not recommend including feature aliases in these tables as this information is dynamically supplied by the $Mapper$ slot (see **Hotgenes object: metadata slots**).

The $designMatrix$ slot requires an R model matrix specifying the comparisons used for differential expression analysis. Additionally, if pairwise comparisons were generated, we recommend adding this to the $contrastMatrix$ slot as a matrix object during object creation. For GSVA, the contrast matrix can be called by the $HotgeneSets()$ function to reproduce the comparisons used.

The $Original\_Object$ slot contains a named list of original unmodified content used to create the Hotgenes object. If a Hotgenes object is created by the $HotgenesDEseq2()$ or $Hotgeneslimma()$ function, this slot will contain a “DESeqDataSet” or “EList" object, respectively.

**Hotgenes object: metadata slots**

Sample-level metadata used for differential expression analysis is stored in the $coldata$ slot as a data frame. This object must have row names that match the column names found in the $Normalize\_Expression$ slot data frames. Any additional variables may be provided here during object creation and will be available for downstream visualizations or clustering.

By default, the $Mapper$ slot is a data frame with a “Features” column that is populated with features extracted from the $Normalize\_Expression$ slot. Additional aliases can be merged to this slot by the “Features” column and will be accessible to Hotgenes functions supporting alias mapping.

**Hotgenes object: auxiliary assays and Omics data slots**

The $Normalize\_Expression$ slot contains normalized Omics data used for differential expression analysis. During object creation, this must be provided in the form of a data frame contained within a named list. Column names reflect sample identities and row names must match feature identities found in the “Feature” columns of the $Output\_DE$ slot.

By default, the $auxiliary\_assays$ slot contains a data frame with a single column called “SampleIDs” that is populated with the row names extracted from the $coldata$ slot during object creation. Additional columns can be merged to this slot by the “SampleIDs” column, if they do not have names shared with features or variables found in $Normalize\_Expression$ or $coldata$ slots.
